# Hypoplasia of dopaminergic neurons by hypoxia-induced neurotoxicity is associated with disrupted swimming development of larval zebrafish

**DOI:** 10.3389/fncel.2022.963037

**Published:** 2022-09-23

**Authors:** Jong-Hyun Son, Amanda K. Gerenza, Gabrielle M. Bingener, Joshua L. Bonkowsky

**Affiliations:** ^1^Department of Biology, Neuroscience Program, University of Scranton, Scranton, PA, United States; ^2^Department of Pediatrics, School of Medicine, Brain and Spine Center, Primary Children’s Hospital, University of Utah, Salt Lake City, UT, United States

**Keywords:** hypoxia, dopaminergic neurons, development, reactive oxygen species (ROS), proapoptotic genes, apoptosis, swimming, hypoxia-recovery (HR)

## Abstract

Hypoxic injury to the developing brain increases the risk of permanent behavioral deficits, but the precise mechanisms of hypoxic injury to the developing nervous system are poorly understood. In this study, we characterized the effects of developmental hypoxia (1% pO_2_ from 24 to 48 h post-fertilization, hpf) on diencephalic dopaminergic (DA) neurons in larval zebrafish and the consequences on the development of swimming behavior. Hypoxia reduced the number of diencephalic DA neurons at 48 hpf. Returning zebrafish larvae to normoxia after the hypoxia (i.e., hypoxia-recovery, HR) induced reactive oxygen species (ROS) accumulation. Real-time qPCR results showed that HR caused upregulation of proapoptotic genes, including *p53* and *caspase3*, suggesting the potential for ROS-induced cell death. With HR, we also found an increase in TUNEL-positive DA neurons, a persistent reduction in the number of diencephalic DA neurons, and disrupted swimming development and behavior. Interestingly, post-hypoxia (HR) with the antioxidant N-acetylcysteine partially restored the number of DA neurons and spontaneous swimming behavior, demonstrating potential recovery from hypoxic injury. The present study provides new insights for understanding the mechanisms responsible for motor disability due to developmental hypoxic injury.

## Introduction

Developmental hypoxia is a significant complication of premature birth. Each year, almost 500,000 infants in the USA and 13 million infants worldwide are born prematurely (defined as birth before 37 gestation weeks) (Beck et al., 2010; Martin et al., 2013). Although survival rates have improved dramatically for premature infants, most premature infants are at elevated risk for developing a neurodevelopmental disorder later in their lives, including attention-deficit disorder, autism, cerebral palsy, motor impairment, depression, epilepsy, and intellectual disability (Martin et al., 2011; Murphy et al., 2017; Purisch and Gyamfi-Bannerman, 2017). In particular, cerebral palsy and other disruptions of motor skills, which have an estimated prevalence in 3 to 4 per 1,000 children in the United States (Boyle et al., 2011; Van Naarden Braun et al., 2016), are associated with hypoxic-ischemic injury and prematurity. Over the past decades, many studies have revealed that developmental hypoxia disrupts multiple aspects of brain development, including cell growth, axon pathfinding, and synapse development (Stevenson et al., 2012; Sharples et al., 2014; Xing et al., 2015; Nalivaeva et al., 2018; Son et al., 2020; Brandon et al., 2022). However, the precise mechanisms of developmental hypoxia leading to disruption of the development of motor behavior, including cerebral palsy, remain poorly understood.

Developmental hypoxia could differentially impact brain development based on the timing, frequency, severity, and duration of the hypoxia (Ducsay et al., 2018). In one study, up to 600 hypoxic episodes per week, each lasting at least longer than 10 s, were reported in premature infants (Martin et al., 2011). Hypoxic episodes that occur during a critical period of brain development, including neurogenesis, differentiation, and when axon and synapse connections are forming, may result in a wide range of pathophysiological outcomes (Ten Donkelaar et al., 2004; Kostovic and Jovanov-Milosevic, 2006; Stiles and Jernigan, 2010). Another critical aspect of hypoxia is the effect on the developmental process occurring during reoxygenation periods after hypoxia, for example, how neurons execute a compensatory accelerated “catch-up process” after returning to normoxia (i.e., reoxygenation) following hypoxia. This catch-up response (i.e., hypoxia-recovery, HR) has been reported to coordinate embryonic/fetal growth and developmental rate in response to changing environmental conditions, including oxygen levels (Azzam and Mortola, 2007; Kamei et al., 2011; Radom-Aizik et al., 2018). Although the “catch-up” response has been found as an evolutionarily conserved mechanism ranging from *Caenorhabditis elegans* to mammalian systems, the precise mechanism of the “catch-up” response in the developing brain is poorly understood (Hales and Ozanne, 2003; Ozanne and Hales, 2004; Saenger et al., 2007).

A potential mediator of adverse effects of hypoxia are reactive oxygen species (ROS), including free radicals like O^⋅–^_2_, OH^⋅^ and non-radicals like H_2_O_2_ and ^1^O_2_ (Valko et al., 2007; Sies and Jones, 2020). Hypoxia reduces aerobic oxidative respiration rates involved in a mitochondrial electron-transport system but increases ROS accumulation and nitric oxide synthase, ultimately leading to cell death through autophagy, apoptosis, and necrosis (Alvarez et al., 2003; Abramov et al., 2007; Kalogeris et al., 2012). Paradoxically, a sudden increase in oxygen after hypoxia (i.e., HR recovery) worsens outcomes because ROS accumulation results in oxidative stress that damages cells (Valko et al., 2007; Chen et al., 2018). However, there is uncertainty regarding the effects of ROS as destructive agents in the context of the nervous system. Evidence shows that neurons utilize ROS as a signaling molecule to regulate neuronal development and function, including growth cone pathfinding and synaptic connectivity/transmission (Munnamalai and Suter, 2009; Massaad and Klann, 2011; Oswald et al., 2018). Therefore, it is interesting to examine the functional characteristics of ROS during central nervous system development under normoxic vs. hypoxic conditions. In particular, studying the effects of hypoxia-induced ROS on the development of the diencephalic dopaminergic system and its regulatory mechanism for locomotor development might bring new insight to the understanding of motor disability related to developmental hypoxic injury.

The tyrosine hydroxylase (TH) positive dopaminergic (DA) nervous system is well-characterized in the vertebrate zebrafish (*Danio rerio*) and serves as a valuable model for studies of hypoxia-associated neurotoxicity in the locomotor development (Son et al., 2016; Bonkowsky and Son, 2018). The first TH^+^ DA neurons are detected between 18 and 20 hpf in the prospective posterior tuberculum (PT), and the mature pattern of DA nuclei and axonal projections that are completed by 4–5 days post-fertilization (dpf) likely corresponds to adult zebrafish DA neurons (McLean and Fetcho, 2004; Kastenhuber et al., 2010; Du et al., 2016). Rink and Wullimann, 2001, 2002 describe seven distinct subpopulations of ventral diencephalic DA groups are defined: DC1, ventral thalamic DA neurons; DC2 and 4, rostral and caudal posterior tubercular DA neurons; DC3, medial hypothalamic DA neurons; DC 5 and 6, hypothalamic DA groups; and DC7, caudal hypothalamic groups. The diencephalic DA neurons and their descending projections to the spinal cord, such as DC4/5 DA neurons, are of particular interest because of their known role in the development of mature adult-like swimming behavior during 5–6 dpf (Lambert et al., 2012; Reimer et al., 2013; Reinig et al., 2017; Haehnel-Taguchi et al., 2018).

In the present study, we characterized the effects of early developmental hypoxia (1% of pO_2_ from 24 to 48 hpf) on the diencephalic DC 4/5 DA neurons, focusing on the direct effects of hypoxia and the indirect “catch-up” response to hypoxia (i.e., HR). We found that hypoxia caused an overall growth restriction of zebrafish larvae observed at 48 hpf immediately following the end of hypoxia. We also found that HR increased the ROS accumulation and upregulated the expression of proapoptotic genes, which might result in hypoplasia of TH^+^ DC4/5 diencephalic DA neurons, subsequent disruption of swimming development, and even a persistent decrease in swimming activity in young adulthood. Additionally, N-acetylcysteine (NAC) treatment, a well-known antioxidant, demonstrated promising effects on recovery in post-hypoxia by restoring the DC4/5 TH^+^ neurons, although the potential mechanism remains to be determined. The present study expands our understanding of the mechanisms of motor disability related to developmental hypoxic injury.

## Materials and methods

### Ethics statement

All zebrafish care and experimental manipulations were approved by the Institutional Animal Care and Use Committee of the University of Scranton and followed *Guide for the Care and Use of Laboratory Animals* (8th Ed., National Research Council).

### Maintenance of adult zebrafish

Adult zebrafish were bred according to standard methods (Westerfield, 2000). Embryos were raised at 28.5°C in E3 embryo medium (5.0 mM NaCl, 0.17 mM KCl, 0.33 mM CaCl_2_, 0.33 mM MgSO_4_, pH 7.0–7.4) with methylene blue, and embryos older than 24 hpf were treated with 0.003% phenylthiourea (PTU) to prevent pigment formation. For immunohistochemistry, embryos were fixed in 4% paraformaldehyde (PFA) in PBS overnight at 4°C, washed briefly in PBS with 0.1% Tween-20, dehydrated stepwise in methanol (MeOH; 30, 50, 70, and 100%), and stored in 100% MeOH at −20°C until use.

### Hypoxia reagents and hypoxia recovery

**FIGURE 1:**
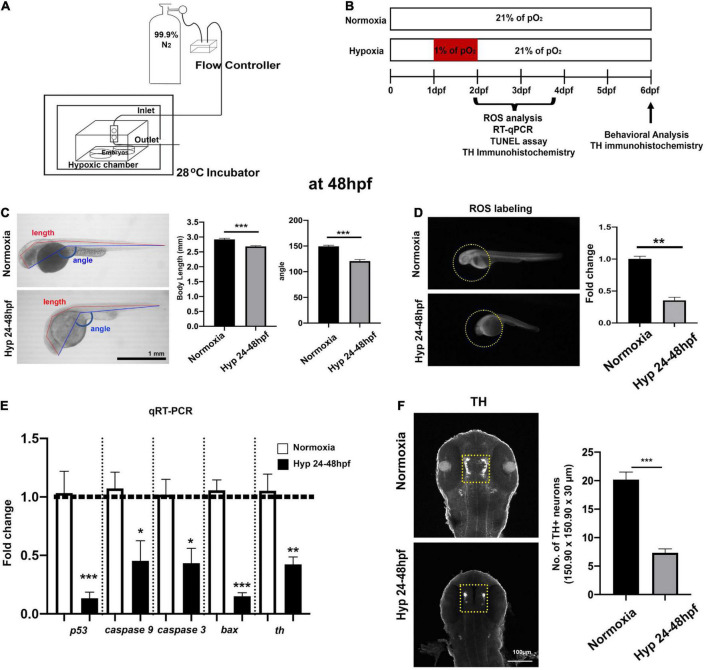
Experimental setup for developmental hypoxia and procedures and the effects of direct hypoxia (Hyp)-induced toxicity at 48 hpf. **(A)** A schematic diagram for the hypoxia setup, **(B)** the timeline for the experimental procedures, **(C)** the whole-body images of zebrafish for the comparison of body length (mm) and head-to-tail angle (HTA) in normoxia (*n* = 12) and hypoxia zebrafish (*n* = 12), **(D)** the ROS production/accumulation in zebrafish head analyzed with Student’s *t*-test and presented as fold changes in hypoxic zebrafish (*n* = 4) compared to controls (*n* = 5), **(E)** the expression of proapoptotic genes (*p53, caspase9, caspase3*, and *bax*) and tyrosine hydroxylase (*Th*) in hypoxic zebrafish (*n* = 6/group, triplicated) corresponding to the expression of reference gene (i.e., *hatn10*) analyzed with Student’s *t*-test and presented by percent changes compared to normoxic controls (*n* = 6/group, triplicated), and **(F)** Z-stack confocal images of normoxic and hypoxic zebrafish (ventral views, rostral to the top), anti-TH immunohistochemistry used to determine the number of TH^+^ neurons in the diencephalic area (150.90 μm × 150.90 μm × 30 μm) in hypoxic zebrafish (*n* = 12) compared to normoxic controls (*n* = 12). For this and in subsequent figures, data are shown as mean ± SEM unless otherwise noted and statistically analyzed with Student’s *t*-test. *p < 0.05, **p < 0.01, and ***p < 0.001.

We followed the previously established hypoxia protocols (Stevenson et al., 2012; Xing et al., 2015; Son et al., 2020). Briefly, embryonic zebrafish were placed in a sealed Plexiglass chamber connected via a controller that monitored and adjusted nitrogen gas flow to a desired 1% pO_2_ set point in an incubator (set as 28°C) (Figure 1A). Previous work had demonstrated that 1% of pO2 hypoxia is non-lethal (Stevenson et al., 2012; Son et al., 2020). Since the equilibration of oxygen partial pressure in water could take several hours, we pre-equilibrated all solutions for at least 4 h before use. We then transferred embryos into the pre-equilibrated solutions, either hypoxia (1% of pO_2_) or normoxia (21% of pO_2_) during ages 24–48 hpf. We terminated hypoxia by returning zebrafish larvae to E3 buffer kept in normoxic conditions (hypoxia recovery, HR). The zebrafish larvae were grown under normoxic conditions for further analysis (Figure 1B). Standard morphological staging was used to help determine age at fixation for analyses.

### Growth and development measurements

Live normoxia control and hypoxia zebrafish larvae at 48 hpf were collected and anesthetized by tricaine. Live imaging was performed using a compound microscope attached Microscope Camera (AmScope, Irvine, CA, USA). We used FIJI (FIJI^1^) segmented line and angle tool to measure means ± standard error of the means (SEM) head-trunk angle (HTA) and body length (defined as the distance from the retina’s center to the end of the tail), both of which are the well-established parameters for determining the developmental stage of a zebrafish embryo (Kamei et al., 2011).

### Tyrosine hydroxylase immunohistochemistry

We visualized the diencephalic DA neurons by immunohistochemistry. Antibodies used were: rabbit polyclonal anti-TH (1:250; Millipore AB152, Burlington, MA, USA) as the primary and Cy-3 anti-rabbit (1:400; Millipore, AP132C, Burlington, MA, USA) as the secondary antibody. The immunohistochemistry for TH was performed as follows: the larval zebrafish were blocked in PBS/1% dimethyl sulfoxide (DMSO)/2% normal goat serum (NGS)/2% bovine serum albumin for 2 h at room temperature (RT), and then incubated in blocking buffer with primary TH antibody overnight at 4°C. The larval zebrafish were washed with PBS/1% DMSO/2% NGS for 6 h at RT and incubated with Cy-3 secondary antibody overnight at 4°C. The larval zebrafish were washed with PSB/1% DMSO/2% NGS and then mounted on the microscopic slides with 70% glycerol. TH images were captured by confocal microscopy and analyzed by imaging software FIJI.

### Measurement of reactive oxygen species generation

The ROS content was examined using a fluorescent probe, 2′-7′dichlorofluorescin diacetate (DCFH-DA; Sigma Aldrich, St. Louis, MO, USA) in live zebrafish larvae at 48, 72, and 96 hpf. Zebrafish larvae were briefly rinsed twice with PBS and incubated with 3 μM DCFH-DA for 2 h in the dark at RT and then washed three times with PBS for 5 min each. The lateral view whole zebrafish body images of each embryo were captured using a microscopic camera (Amscope HDMI Color CMOS C-mount Camera, Amscope, Irvine, CA, USA) attached to an Olympus SZ51 stereomicroscope (Olympus, Barrington, NJ, USA), illuminated by NIGHTSEA (Lexington, MA, USA) fluorescence illumination system with a royal blue excitation cube. For higher magnification (3×) focusing on the head only, each live zebrafish larvae were mounted with low-melt agarose (1.5%) onto the depressed microscopic slide. The ventral/dorsal view images of each larva were captured using an Amscope Digital camera (5MP USB3.0 High-Speed Color CMOS C-mount Microscope Camera, Amscope, Irvine, CA, USA) attached to an Olympus SZX7 stereomicroscope (Olympus, Barrington, NJ, USA), illuminated by X-Cite120 LED mini (Excelitas Technologies, Waltham, MA, USA) with the fluorescence light power between 8 and 12%. The captured ROS images were analyzed by imaging software FIJI. Different density was measured and presented as a fold change compared to control.

### Real-time qPCR analysis for proapoptotic genes and *tyrosine hydroxylase*

Total RNA was extracted from 12 zebrafish larvae at 48, 72, and 96 hpf, using Zymo quick RNA Tissue/Insect Kit (Zymo Research, Irvine, CA, USA) following the manufacturer’s guidelines. The RNA quality and quantity were confirmed by a Nanodrop 2000 Spectrophotometer (ThermoFisher Scientific, Waltham, MA, USA). The cDNA was prepared using 100–200 ng RNA from each sample with a LunaScript RT reagent kit (New England BioLabs, Ipswich, MA, USA). The 20 μl real-time polymerase chain reaction mixtures included 10 μl Luna Universal qPCR master mix (New England Biolab, Ipswich, MA, USA), 1 μl of 50 nM primers, 1 μl cDNA template, 8 μl of DNAase/RNAase free water; the reactions were performed using Chai Open qPCR (Chaibio, Santa Clara, CA, USA). Real-time PCR thermal cycling was set as follows: 95°C for 1 min and then 40 cycles of 95°C for 15 s followed by 60°C for 30 s. The melting curve analysis was performed at 95°C for 30 s followed by 60°C for 1 min, gradual heating to 95°C for 30 s at a gradual ramp-rate 0.1°C/s, followed by cooling to 4°C for 1 min to verify primer specificity in each assay. The 2^–ΔΔCt^ method calculated the relative gene expression was presented as a percent change compared to the normoxic control. The primer sequences of *p53*, *caspase9*, *caspase3*, *bax*, and *th* are shown in Supplementary Table 1. The *hatn10* repetitive element was used as an internal control gene (Vanhauwaert et al., 2014). The 2^–ΔΔCt^ (i.e., cycle threshold indicating how many cycles a machine needed to detect the fluorescent signal of targeted genes) represented a normalized, relative gene expression value to the endogenous reference gene, the *hatn10* gene, and the data were presented as a fold change compared to the normoxic control.

### Transferase dUTP nick-end labeling assay and tyrosine hydroxylase immunohistochemistry

Terminal deoxynucleotidyl transferase dUTP nick-end labeling (TUNEL) staining was performed on whole-mount larvae and then followed by immunohistochemistry to visualize the diencephalic DA neurons (Son et al., 2016, 2020). Briefly, after standard fixation of larval zebrafish with 4% of PFA and dehydration in 100% MeOH, larvae were rehydrated stepwise into PBS with 0.1% Tween 20 (PBST), permeabilized with 10 mg/ml Proteinase K in PBST at 28°C, washed twice with PBST, re-fixed for 20 min with 4% PFA, and washed again with PBST. Subsequently, 75 ml of equilibration buffer was added to the larvae for 1 h and then removed and replaced with 55 ml of “working-strength” (per Apoptosis Detection kit instructions; Millipore, Burlington, MA, USA) terminal deoxynucleotidyl transferase enzyme overnight at 37°C. Eppendorf tubes were sealed with Parafilm to prevent drying out the larvae. The end-labeling reaction was stopped by washing the embryos three times for 15 min each with 2 ml of the stop/wash buffer, followed by three 5 min washes with PBS. Then 65 ml of Working-strength sheep anti-digoxigenin rhodamine antibody (Millipore, Burlington, MA, USA) was added to the embryos overnight at 4°C. Diencephalic DA neurons were then visualized by immunohistochemistry, as described above. The TUNEL and TH images were captured by confocal microscopy and analyzed by imaging software FIJI and presented as Means ± SEM.

### Spontaneous swimming behavior analysis

Using a video analysis software program, larval zebrafish behavior analysis was performed at 6 dpf in 96-well round-bottom plates (Noldus EthoVision XT16, Leesburg, VA, USA). For spontaneous swimming observation, animals were transferred to a 96-well black plate for 30 min acclimations before recording behavior. The spontaneous behavior, including swimming distance, swimming velocity, and frequency of moving, was measured for a total of 60 min, including under alternate dark (3 × 10 min) and light (3 × 10 min) conditions.

Young adult zebrafish (1.5-month-old) after developmental hypoxia were also used to determine their spontaneous swimming behavior, which was measured for a total of 10 min each, including dark (10 min) and light (10 min) in an 8.5 cm wide × 6.5 cm long deep jar. Housing water was filled up to 3 cm height during recording. The movies were used to analyze their swimming behavior (i.e., swimming distance, swimming velocity, and frequency of moving under dark and light) using the video analysis software (Noldus Ethovision XT16, Leesburg, VA, USA) and presented as Means ± SEM.

### N-acetylcysteine treatment under hypoxia and hypoxia-recovery

Zebrafish embryos were manually dechorionated at 24 hpf and transferred into the pre-equilibrated hypoxic solutions (PTU/E3) containing the 2 μg/ml of NAC and 0.1% DMSO. The zebrafish larvae were incubated for 24 h under 1% pO2 hypoxic conditions. The 0.1% DMSO in the hypoxic solution (PTU/E3) was used as a control. At 48 hpf, the level of ROS, the number of TH neurons, and proapoptotic gene expression analysis were determined as described above. The experimental schematic diagram is shown in Figure 4A.

To further determine the potential effects of NAC by HR after hypoxia, each normoxic and hypoxic group of zebrafish was further separated into the NAC treatment and control group. We exposed zebrafish larvae to the embryonic solution (PTU/E3) containing 2 μg/ml of NAC/0.1% DMSO under normoxic conditions from 48 to 96 hpf, whereas we used the PTU/E3 containing 0.1% DMSO as a control. At 96 hpf, we collected the zebrafish larvae and determined the number of TH neurons, ROS level, proapoptotic gene level, and cell death by TUNEL/TH immunohistochemistry. The detailed experimental paradigm is depicted in Figure 5A.

### Image and statistical analysis

Z-stack compiled confocal images by FIJI (see text footnote 1) were used to analyze TUNEL^+^ and TH^+^ DA neurons. The TUNEL^+^ signal was determined by the “Analyze Particles” function in FIJI. Briefly, we cropped the Z-stack confocal images containing the diencephalon (e.g., 150.90 μm × 150.90 μm × 30 μm for 48 hpf; 174.22 μm × 174.22 μm × 30 μm for 72 and 96 hpf), set the threshold for the signals, and then measured the TUNEL positive signals using the “Analyze Particles” function with the size parameter (micron^2^) as 5.00–100 and the circularity as 0–1. The number of TH^+^ DA neurons was manually counted. Images were coded so that all subsequent analyses were conducted by an experimenter blinded to the treatment groups.

Numerical data are presented as Means ± SEM, and the data of ROS and qRT-PCR are presented as a fold change. All dependent measures across hypoxia and normoxia were completed with Student’s *t*-test. The NAC treatment data were analyzed with two-way ANOVA followed by Tukey’s *post hoc* analysis using GraphPad Prism 9.0 (San Diego, CA, USA). Overall, statistical significance was set at *p* < 0.05.

## Results

### Growth restriction and delayed development of dopaminergic neurons at 48 h post-fertilization after hypoxia

To determine the direct effects of hypoxia on the development of zebrafish larvae, we measured body length and head-tail angle (HTA) at 48 hpf (Figure 1C). The overall growth restriction in hypoxic zebrafish was evidenced by reduction both in the body length [Normoxia, *n* = 12, 2.92 ± 0.01 mm; Hypoxia, *n* = 12, 2.68 ± 0.02 mm; *t*(22) = 5.4 *p* < 0.001] and in HTA [Normoxia, *n* = 12, 149.4 ± 2.16°; Hypoxia, *n* = 12, 120.6 ± 3.05; *t*(22) = 7.7, *p* < 0.001]. Interestingly, the level of ROS was barely visible in the hypoxic zebrafish compared to normoxic controls, indicating the lack of aerobic cellular respiration that would typically produce the ROS [*t*(7) = 10, *p* < 0.01] (Figure 1D). In addition, we measured the expression of proapoptotic genes (e.g., *p53, caspase 9, caspase 3*, and *bax*) and *tyrosine hydroxylase (th)* at 48 hpf. We found the reduced expression of *P53* [*t*(4) = 4.669, *p* < 0.001], *Caspase 9* [*t*(4) = 2.810, *p* < 0.05], *Caspase 3* [*t*(4) = 3.226, *p* < 0.05], *Ba*x [*t*(4) = 9.659, *p* < 0.001], and *tyrosine hydroxylase (Th)* [*t*(10) = 4.042, *p* < 0.01] in hypoxic vs. normoxic (Figure 1E). Further, we found that there was a significantly reduced number of TH^+^ neurons [Normoxia, 20.2 ± 1.3; Hypoxia, 7.3 ± 0.7 in 150.90 μm × 150.90 μm × 30 μm; *t*(22) = 8.5, *p* < 0.01], indicating the developmental hypoxia restricted the development of TH^+^ DA neurons (Figure 1F). Interestingly, we did not observe an increased level of apoptotic markers in hypoxic zebrafish compared to controls at 48 hpf (data not shown). Overall, hypoxia resulted in a significant growth restriction, reduced ROS levels, increased expression of proapoptotic genes, and reduced number of TH^+^ DA neurons in hypoxic zebrafish, suggesting hypoxia-induced developmental delay.

### Hypoxia-recovery does not fully restore the development of dopaminergic neurons at 72 and 96 h post-fertilization

**FIGURE 2:**
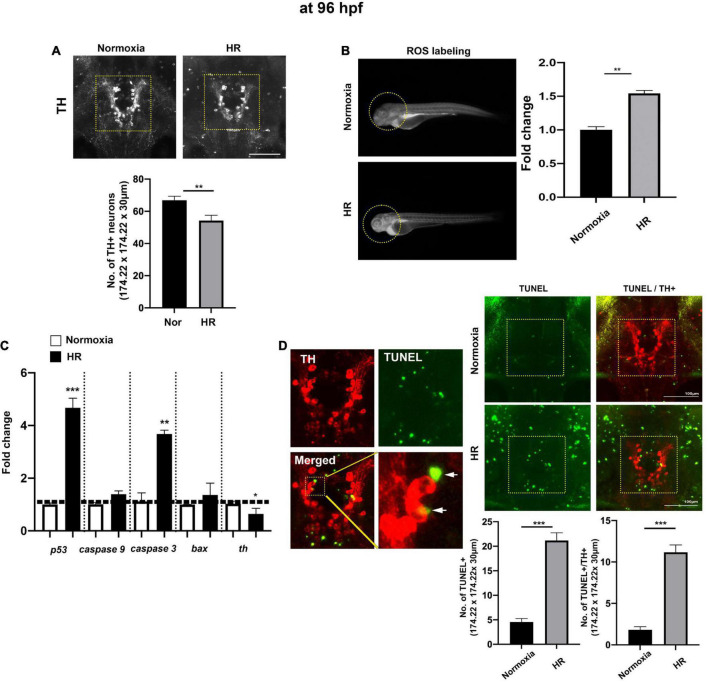
The indirect “catch-up” response to hypoxia (i.e., HR) at 96 hpf following hypoxia. **(A)** The Z-stack confocal images (ventral views, rostral to the top) were used to determine the maximum intensity projections and the number of TH^+^ neurons in the diencephalic area (170.22 μm × 170.22 μm × 30 μm) in normoxic (*n* = 11) and HR zebrafish (*n* = 12) analyzed with Student’s *t*-test and shown as Means ± SEM, **(B)** the ROS production/accumulation in zebrafish head analyzed with Student’s *t*-test and presented as fold changes in HR zebrafish (*n* = 5) compared to controls (*n* = 5), **(C)** the expression of proapoptotic genes (*p53, caspase9, caspase3*, and *bax*) and tyrosine hydroxylase (*th*) in normoxic and HR zebrafish (*n* = 6/group, triplicated) corresponding to the expression of reference gene (hatn10) analyzed with Student’s *t*-test and presented by percent changes compared to normoxic controls (*n* = 6/group, triplicated), **(D)** the magnified TH^+^, TUNEL^+^, and merged TH^+^/TUNEL^+^ images and arrows indicate TUNEL^+^ and TH^+^ neurons. The TUNEL labeling in green and anti-TH in red (174.22 μm × 174.22 μm × 30 μm) in HR zebrafish (*n* = 11) compared to normoxic zebrafish larvae (*n* = 12). *p < 0.05, **p < 0.01, and ***p < 0.001.

The “catch-up” cellular response by HR was examined at 72 hpf (Supplementary Figure 1) and 96 hpf (Figure 2). At 72 hpf, we observed the increased ROS [*t*(8) = 6.1, *p* < 0.01, Supplementary Figure 1A] and decreased numbers of TH^+^ DA neurons in hypoxic zebrafish with HR compared to normoxic controls [*t*(19) = 13, *p* < 0.001, Supplementary Figure 1B]. The expression of proapoptotic genes, such as *p53*, *caspase 9*, *caspase3*, and *bax*, were similar level to normoxic controls (Supplementary Figure 1C). In contrast, there was an increased number of cells with phospho-histone 3 (PH3) staining in the diencephalic area. Collectively, the results indicate the accelerating developmental process by HR at 72 hpf in hypoxic zebrafish [*t*(12) = 2.4, *p* < 0.05, Supplementary Figure 1D]. However, we also observed a decrease in the number of GFP^+^ DA neurons driven by the dopaminergic transcription factor, otpb. A demonstrating the persistent reduction in the number of DA neurons by HR [*t*(12) = 2.6, *p* < 0.05, Supplementary Figure 1D]. Our results suggest that the accelerating “catch-up” process by HR did not restore the DA neurons.

At 96 hpf, the confocal image analysis of TH^+^ DA neurons indicates that HR did not fully restore the development of DA neurons, evidenced by the reduced number of TH^+^ DA in hypoxic zebrafish with HR (HR from now on) compared to the normoxic control [*t*(21) = 3.0, *p* < 0.01, Figure 2A]. The ROS level was significantly increased in HR zebrafish at 96 hpf [*t*(8) = 8.4, *p* < 0.01], suggesting that HR increased the production/accumulation of ROS in hypoxic zebrafish larvae (Figure 2B). The proapoptotic genes, such as *p53* and *caspase 3*, were likewise increased in HR zebrafish (Figure 2C): *p53* expression was an approximately four-fold [*t*(4) = 9.914, *p* < 0.001], and *caspase 3* was increased three-fold [*t*(4) = 7.283, *p* < 0.01] vs. normoxic zebrafish. On the other hand, a *th* expression decreased in hypoxic zebrafish by HR [*t*(9) = 4.113, *p* < 0.01] (Figure 2C). We then examined the number of TH^+^ DA neurons with apoptotic markers (Figure 2D) and found a significantly increased number of TUNEL^+^ cells in the diencephalic areas in the HR zebrafish [Normoxia, 4.5 ± 0.7; HR, 21.2 ± 1.6 in 174.22 μm × 174.22 μm × 30 μm; *t*(21) = 9.283, *p* < 0.01, Figure 2D]. Further, we also observed the increased number of neurons which were TUNEL^+^ and TH^+^ in HR zebrafish [Normoxia, 1.8 ± 10.4; HR, 11.2 ± 0.9; *t*(21) = 9.318, *p* < 0.01, Figure 2D]. Our findings suggest that HR in hypoxic zebrafish might cause hypoplasia of TH^+^ diencephalic DA neurons, associated with increased apoptotic signals that result from the accumulated ROS by HR (Figure 2). More detailed data about the number of TH^+^ DA neurons at different developmental stages are shown in Supplementary Table 2.

### Effects of hypoxia on the number of DC4/5 dopaminergic neurons and spontaneous swimming development at 6 days post-fertilization and young adult

**FIGURE 3:**
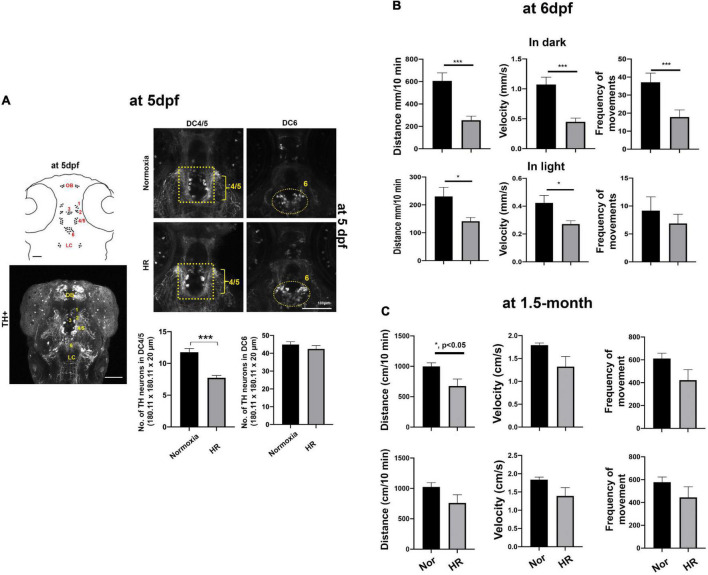
**(A)** A schematic drawing (left, top panel) and Z-stack confocal image (left, bottom panel) of diencephalic TH^+^ DA neurons at 5 dpf, including in DC1, DC2, DC3, DC4/5, and DC6. **(A)** Z-stack confocal images of the anti-TH immunohistochemistry were analyzed in the DC4/5 and DC 6 areas (180.11 μm × 180.11 μm × 20 μm) in HR zebrafish (*n* = 23) compared to normoxic controls (*n* = 20) analyzed with Student’s *t*-test and presented as Means ± SEM. ***p < 0.001. **(B)** Developmental hypoxia results in disrupted swimming maturation at 6 dpf. Data plots (Means ± SEM) show the averaged swimming distance, velocity, and frequency of movements *n* the dark (3 × 10 min) and in light (3 × 10 min) in HR larvae (*n* = 24) compared to normoxic controls (*n* = 24). Student’s *t*-test, ***p < 0.001, and *p < 0.05. **(C)** Developmental hypoxia causes reduced swimming distance in dark in 1.5-month-old young zebrafish (hypoxia, *n* = 6 vs. normoxia, *n* = 6). ***p < 0.001, *p < 0.05.

We further characterized the spatial distribution of diencephalic DA neurons using whole-mount TH immunohistochemistry. Consistent with previous studies (Rink and Wullimann, 2001, 2002), our TH immunohistochemistry also showed the majority of TH^+^ DA neurons in diencephalic areas with seven diencephalic clusters (DC), including DC1, DC2, DC3, DC4, DC5, DC6, and DC7 at 5 dpf (Figure 3A). In particular, we focused on the DC4/5 TH^+^ neurons as they project their axons to the spinal cord and regulate the maturation of adult-like swimming during development (Lambert et al., 2012; Son et al., 2020). We observed that there was a significant decrease in the DC4/5 TH^+^ neurons at 5 dpf in HR zebrafish (7.7 ± 0.37 in 180.11 μm × 180.11 μm × 20 μm) compared to normoxic controls [11.8 ± 0.59 in 180.11 μm × 180.11 μm × 20 μm, *t*(32) = 5.746. *p* < 0.01, Figure 3A]. In contrast, there were no statistically different numbers of TH^+^ neurons in DC6 in HR zebrafish (41.9 ± 1.22, in 180.11 μm × 180.11 μm × 20 μm) compared to the neurons in controls [40.8 ± 1.45, *t*(32) = 0.5579, *p* = 0.64, Figure 3A], suggesting different developmental responses in subpopulations of TH^+^ DA neurons against the hypoxic-injury (Figure 3A).

**FIGURE 4:**
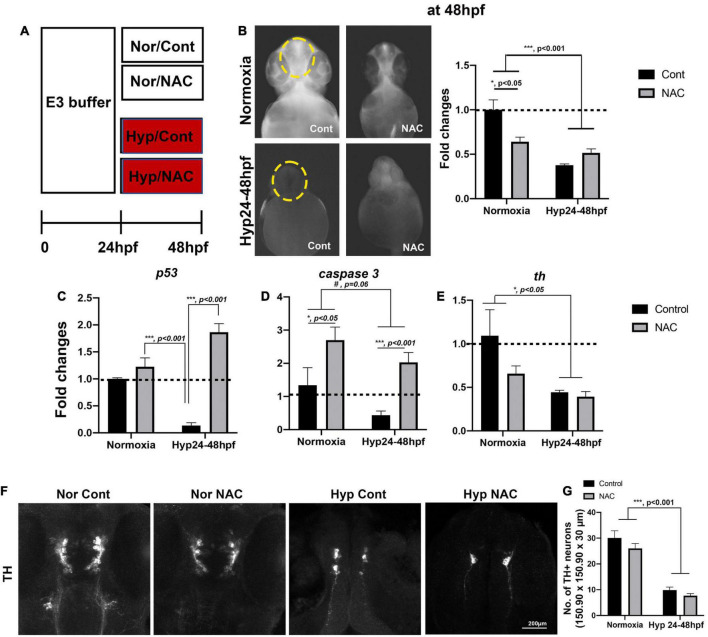
**(A)** A schematic diagram for NAC treatment procedures with normoxia (Nor) and hypoxia (Hyp) and the effects of NAC vs. control (Cont) against direct-hypoxic injury at 48 hpf. **(B)** ROS production/accumulation in zebrafish head at 48 hpf in normoxic zebrafish with control (*n* = 6), normoxic zebrafish with NAC (*n* = 6), hypoxic zebrafish with control (*n* = 3), and hypoxic zebrafish with NAC (*n* = 5) analyzed by two-way ANOVA followed by Tukey’s *post hoc* test and presented as fold change compared to normoxic control. **(C–E)** Different expression of proapoptotic genes (*p53, caspase9, caspase3*, and *bax*) by HR, including normoxic control (*n* = 6/group, triplicated) and normoxic NAC (*n* = 6/group, triplicated), hypoxic control (*n* = 6/group, triplicated), and hypoxic NAC (*n* = 6/group, triplicated) analyzed by two-way ANOVA followed by Tukey’s *post hoc* test and presented by percent changes. **(F)** The Z-stack, confocal images, and **(G)** corresponding cell counts of anti-TH at 48 hpf (150.90 μm × 150.90 μm × 30 μm) in normoxic control (*n* = 5), normoxic NAC (*n* = 6), and hypoxic control (*n* = 5), and hypoxic NAC (*n* = 6) analyzed by two-way ANOVA followed by Tukey’s *post hoc* test and presented by Means ± SEM. *p < 0.05, ***p < 0.001, and ^#^*p* = 0.06.

**FIGURE 5:**
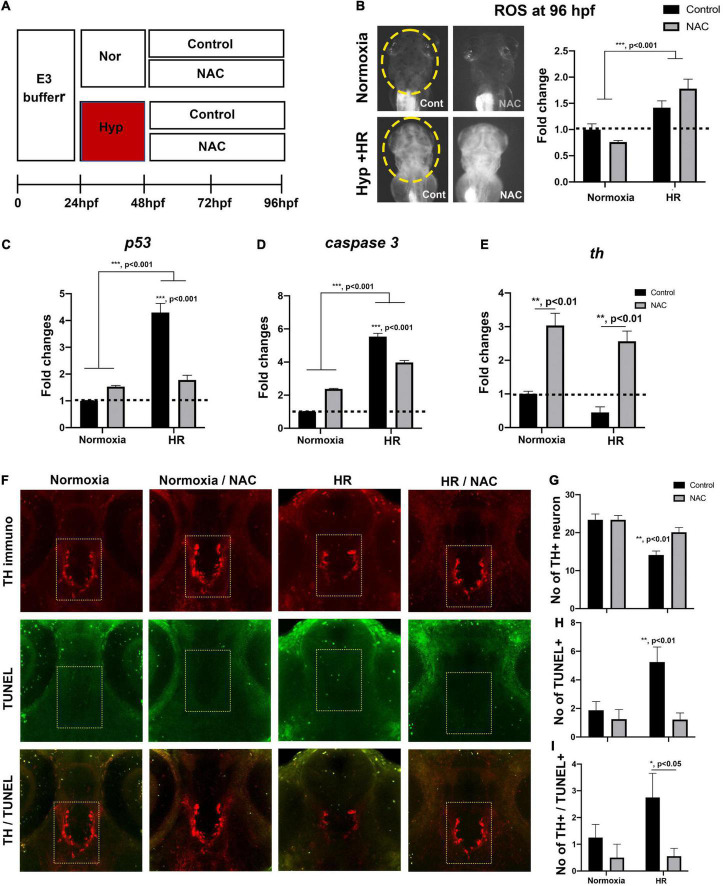
**(A)** A schematic diagram for NAC treatment procedures by HR and the effects of NAC with reoxygenation against the hypoxic injury with HR 96 hpf. **(B)** ROS production/accumulation in zebrafish head at 96 hpf in normoxic control (*n* = 16), normoxic NAC (*n* = 17), HR control (*n* = 14), and HR NAC (*n* = 14) analyzed by two-way ANOVA followed by Tukey’s *post hoc* test and presented fold changes compared to normoxic control. **(C–E)** Different expression of proapoptotic genes (*p53, caspase9, caspase3*, and *bax*) by HR, including HR NAC (*n* = 6/group, triplicated) and HR control (*n* = 6/group, triplicated), normoxic NAC (*n* = 6/group, triplicated), and normoxic control (*n* = 6/group, triplicated) analyzed by two-way ANOVA followed by Tukey’s *post hoc* test and presented by percent changes. **(F)** The Z-stack, confocal images of TUNEL^+^ and the number of **(G)** anti-TH, **(H)** TUNEL^+^, and **(I)** anti-TH/TUNEL^+^ at 96 hpf (174.22 μm × 174.22 μm × 30 μm^3^) in normoxic control (*n* = 5), normoxic NAC (*n* = 6), and HR control (*n* = 5), and HR NAC (*n* = 6) analyzed by two-way ANOVA followed by Tukey’s *post hoc* test. *p < 0.05, **p < 0.01, and ***p < 0.001.

To explore the effects of hypoxia on swimming development, we examined the spontaneous swimming behavior in larval zebrafish at 6 dpf because the developmental switch to the matured swimming pattern coincides with the swimming bladder development between 5 and 5 dpf (Buss and Drapeau, 2001; Lambert et al., 2012). We found a significant effect of hypoxia on the spontaneous swimming behavior, including reduced swimming distance in dark [*t*(46) = 4.3, *p* < 0.01] and in light [*t*(46) = 2.5, *p* < 0.05]; velocity both in dark [*t*(46) = 4.6, *p* < 0.01] and in light [*t*(46) = 2.6, *p* < 0.05]; and frequency of moving in dark [*t*(46) = 3.0, *p* < 0.01]. These findings suggest that developmental hypoxia significantly disrupts the swimming maturation of larval zebrafish (Figure 3B). Further, we have examined the spontaneous swimming behavior of 1.5-month-old zebrafish exposed to the developmental hypoxia, demonstrating there was a persistently reduced swimming behavior later in adulthood, including the swimming distance [*t*(10) = 2.496, *p* < 0.05] and decreased velocity [*t*(10) = 2.102, *p* = 0.06] in the dark. Although the frequency of movements in dark and swimming distance and velocity in light did not reach the statistically significant level, there was a consistent trend of reduced swimming distance, velocity, and frequency of movements in hypoxic zebrafish (Figure 3C). The details of swimming behaviors are shown in Supplementary Table 3.

### Effects of N-acetylcysteine on developing zebrafish larvae with hypoxia at 48 h post-fertilization

To evaluate the potential effects of NAC against direct hypoxic injury, we exposed zebrafish larvae to the PTU/E3 buffer containing 2 μg/ml of NAC/0.1% DMSO with hypoxic vs. normoxic conditions (Figure 4A); NAC was omitted from the buffer as control. In hypoxia-treated zebrafish, ROS levels decreased overall compared to normoxic zebrafish. Two-way ANOVA on the ROS level revealed the main effects of hypoxic treatment (*F*_1_,_16_ = 18.63, *p* < 0.001), but NAC treatment did not affect the magnitude of ROS decrease (*F*_1_,_16_ = 1.97, *p* = 0.29). However, there were significant interactions (Hypoxia × NAC, *F*_1_,_16_ = 6.74, *p* = 0.019, Figure 4B). In contrast, Tukey’s *post hoc* comparison revealed that the ROS level with NAC treatment was significantly decreased in the normoxic zebrafish (*p* < 0.05).

The expression of proapoptotic genes, including *p53* and *caspase 3*, and the rate-limiting enzyme of dopamine synthesis, *tyrosine hydroxylase (Th)*, was determined by qRT-PCR. Two-way ANOVA on the *p53* expression revealed the main effects of NAC treatment with normoxic fish (*F*_1_,_8_ = 67.97, *p* < 0.001) but no main effects of hypoxic treatment (*F*_1_,_8_ = 0.92, *p* = 0.37); there were significant interactions (Hypoxia × NAC, *F*_1_,_8_ = 40.44, *p* < 0.001, Figure 4C). Tukey’s *post hoc* comparison revealed that the *p53* expression was significantly increased in the hypoxic zebrafish treated by NAC (*p* < 0.001), but not in normoxic controls (*p* = 0.5694). In addition, there was a noticeable but not statistically significant decrease in the expression of *caspase 3* due to hypoxia (*F*_1_,_8_ = 4.62, *p* = 0.06), and increase in *caspase 3* with NAC treatment for both normoxia and hypoxia (*F*_1_,_8_ = 16.30, *p* < 0.01), but no significant interactions (Hypoxia × NAC, *F*_1_,_8_ = 0.107, *p* = 0.75, Figure 4D). Finally, the analysis of *th* expression showed that hypoxia decreased *th* (*F*_1_,_8_ = 8.319, *p* < 0.05), no effects of NAC treatment (*F*_1_,_8_ = 2.362, *p* = 0.162) and no interaction (Hypoxia × NAC, *F*_1_,_8_ = 1.491, *p* = 0.25, Figure 4E). Overall, the NAC treatment in hypoxic zebrafish increased the *p53* and *caspase 3* expression, but not the *th* expression at 48 hpf immediately following hypoxia.

We further examined the number of TH^+^ DA neurons to determine the potential effects of NAC against the direct effects of hypoxia. Two-way ANOVA revealed that hypoxia reduced the number of neurons (*F*_1_,_18_ = 113.8, *p* < 0.001), but no main effects of NAC treatment (*F*_1_,_18_ = 2.884, *p* = 0.106) and no interaction (Hypoxia × NAC, *F*_1_,_18_ = 0.2672, *p* = 0.61, Figures 4F,G). Thus, the NAC treatment under hypoxic conditions did not restore the development of TH^+^ DA neurons at 48 hpf.

### Effects of N-acetylcysteine on developing zebrafish larvae with HR at 96 h post-fertilization

We treated zebrafish larvae with 2 μg/ml of NAC/0.1% DMSO after hypoxia from 48 to 96 hpf to further evaluate the potential effects of NAC against post-hypoxia (HR) (Figure 5A). Two-way ANOVA showed that HR increased overall ROS levels (*F*_1_,_57_ = 36.65, *p* < 0.001), but no significant NAC treatment effect (*F*_1_,_57_ = 0.282, *p* = 0.59); however, there were significant interactions (HR × NAC, *F*_1_,_57_ = 6.426, *p* < 0.05, Figure 5B). Our Tukey’s *post hoc* comparison revealed that the ROS level with NAC treatment in HR was significantly increased compared to the normoxic control (*p* < 0.001) and normoxic NAC (*p* < 0.001). In contrast, the ROS level was significantly decreased in normoxic NAC compared to HR control (*p* < 0.01). Collectively, our data suggest a dichotomous effect of NAC on the ROS between normoxic vs. hypoxic zebrafish with HR (Figure 5B).

The expression of proapoptotic genes, including *p53* and *caspase 3*, and *th* was also determined by qRT-PCR at 96 hpf (Figures 5C–E). Two-way ANOVA showed that the HR-induced increase *p53* in expression (*F*_1_,_8_ = 84.15, *p* < 0.001), was abolished by NAC treatment (*F*_1_,_8_ = 26.40, *p* < 0.001) with a significant interaction (HR × NAC, *F*_1_,_8_ = 62.10, *p* < 0.001, Figure 5C). Tukey’s *post hoc* comparison revealed that the significantly increased *p53* expression was only observed in HR control compared to normoxic control (*p* < 0.001), normoxic NAC (*p* < 0.001), and HR NAC (*p* < 0.001), suggesting that the NAC treatment inhibited the increased the expression of *p53* proapoptotic gene in HR control zebrafish (Figure 5C). The expression of *caspase 3* also similarly affected HR (*F*_1_,_8_ = 672.5, *p* < 0.001) and significant interactions with NAC treatment (*F*_1_,_8_ = 154.0, *p* < 0.001, Figure 5D). Tukey’s *post hoc* comparison also revealed that the *caspase3* expression was significantly increased in HR NAC (*p* < 0.001), further suggesting that the NAC treatment reduced the expression of *caspase3* proapoptotic gene in HR zebrafish (Figure 5D). In contrast, expression *th* increased with NAC treatment (*F*_1_,_8_ = 67.98, *p* < 0.001), but was unaffected by HR (*F*_1_,_8_ = 4.167, *p* = 0.08), with no interaction (HR × NAC, *F*_1_,_8_ = 0.029, *p* = 0.86, Figure 5E). Overall, the NAC treatment with HR reduced the *p53* and *caspase 3* expression and increased *th* expression in hypoxic zebrafish at 96 hpf (Figures 5C–E).

We also determined the TH^+^ number of DA neurons in hypoxic zebrafish exposed to NAC at 96 hpf, demonstrating that HR significantly decreased number of TH^+^ immunoreactive neurons (*F*_1_,_29_ = 25.2, *p* < 0.001), main effects of NAC treatment (*F*_1_,_29_ = 5.78, *p* < 0.05), with a significant interaction (HR × NAC, *F*_1_,_29_ = 5.78, *p* < 0.05, Figures 5F,G). Tukey’s *post hoc* comparison also revealed that the number of TH^+^ DA neurons was significantly decreased in only HR control compared to the normoxic control (*p* < 0.001), normoxic NAC (*p* < 0.001), and HR NAC (*p* < 0.01), suggesting that the NAC treatment potentially restored the development of TH^+^ DA neurons in hypoxic zebrafish larvae (Figure 5G). Additionally, we also determined the number of TUNEL^+^ cells. Our data also revealed that the NAC treatment reduces cell death in HR (*F*_1_,_29_ = 10.46, *p* < 0.01) with a significant interaction (HR × NAC, *F*_1_,_29_ = 5.59, *p* < 0.05, Figure 5H). Tukey’s *post hoc* comparison also revealed that the number of TUNEL^+^ cells was significantly increased in only hypoxic control compared to the normoxic control (*p* < 0.05), normoxic NAC (*p* < 0.01), and hypoxic NAC (*p* < 0.01), suggesting that the NAC treatment reduced the number of TUNEL^+^ cells in hypoxic zebrafish larvae (Figure 5H). We also determined the TUNEL^+^/TH^+^ DA neurons in hypoxic zebrafish at 96 hpf. Our data revealed that the NAC treatment reduced cell death in TH^+^ neurons (*F*_1_,_29_ = 6.529, *p* < 0.05) but no main effects of HR (*F*_1_,_29_ = 1.822, *p* = 0.18) and no significant interactions (HR × NAC, *F*_1_,_29_ = 1.571, *p* = 0.22, Figure 5I). Together, our results revealed that NAC promotes the development of TH^+^ DA neurons via an independent ROS-mediated inhibition of proapoptotic pathways (Figures 5F–I).

### Effects of N-acetylcysteine on developing zebrafish larvae swimming behavior at 6 days post-fertilization

**FIGURE 6:**
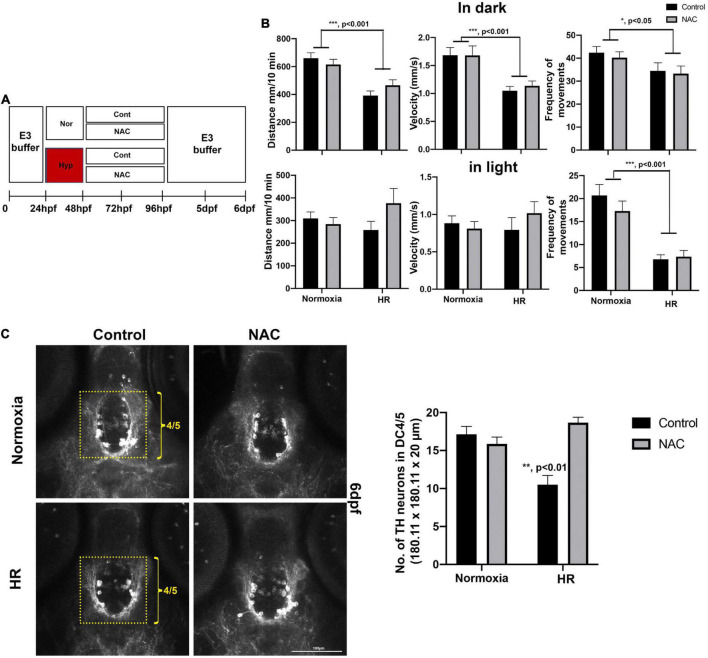
**(A)** A schematic diagram for NAC treatment procedures with HR and the effects of NAC on swimming development and DC4/5 TH^+^ neurons at 6 dpf. **(B)** Behavioral data in normoxic control (*n* = 72), in normoxic NAC (*n* = 70), HR control (*n* = 51), and HR NAC (*n* = 64) were analyzed by two-way ANOVA followed by Tukey’s *post hoc* test. **(C)** The Z-stack, confocal images of anti-TH at 6 dpf in the DC4/5 area (180.11 μm × 180.11 μm × 20 μm) in normoxic control (*n* = 6), normoxic NAC (*n* = 8), and HR control (*n* = 6), and HR NAC (*n* = 12) analyzed by two-way ANOVA followed by Tukey’s *post hoc* test. *p < 0.05, **p < 0.01, and ***p < 0.001.

The NAC with HR did not restore the swimming development (Figure 6). Our two-way ANOVA revealed that hypoxia reduced in swimming distance in dark (*F*_1_,_248_ = 29.12, *p* < 0.001, Figure 6B), swimming velocity in dark (*F*_1_,_248_ = 19.57, *p* < 0.001, Figure 6C), frequency of movements in dark (*F*_1_,_248_ = 6.15, *p* < 0.05) and frequency of movements in light (*F*_1_,_248_ = 35.42, *p* < 0.001, Figure 6B); there were no main effects of NAC and no significant interactions observed in HR NAC zebrafish (Figure 6B). The detailed swimming behaviors are shown in Supplementary Table 4. Once the behavioral analysis was completed, we further examined the TH^+^ DA neurons with the zebrafish at 6 dpf. We found the NAC effect in DC4/5 TH^+^ DA neurons in HR NAC zebrafish (Figure 6C). Two-way ANOVA on the TH^+^ neurons revealed that the decrease in TH^+^ neurons numbers due to hypoxia (*F*_1_,_29_ = 4.034, *p* < 0.05), were ameliorated with NAC treatment (*F*_1_,_29_ = 12.94, *p* < 0.001), with a significant interaction (Hypoxia × NAC, *F*_1_,_29_ = 24.21, *p* < 0.001, Figure 6C). Tukey’s *post hoc* analysis showed that there was a decrease in the number of TH^+^ DA neurons in hypoxic control compared to the normoxic control (*p* < 0.001), normoxic NAC (*p* < 0.01), and HR NAC (*p* < 0.001), suggesting that the NAC treatment might protect the development of TH^+^ DA neurons in hypoxic zebrafish with HR.

## Discussion

We have investigated the effects of early developmental hypoxia on the diencephalic DA neurons in larval zebrafish, focusing on a direct hypoxia-induced cellular response and the indirect catch-up response by HR. In the present study, we demonstrate that developmental hypoxia (24–48 hpf 1% of pO_2_) directly restricts the growth of zebrafish larvae, in particular, the delayed development of DA neurons (Figure 1). Additionally, HR following hypoxia is associated with a chronic disruption of DA neurons, and with impaired swimming behavior (Figures 2, 3). The NAC treatment did not prevent the direct hypoxia-induced growth restriction or development of DA neurons (Figure 4). However, the NAC treatment during HR had promising effects on the recovery, although the precise mechanism remains to be determined (Figures 4–6).

The direct hypoxia-induced growth restriction was quite pronounced at 48 hpf immediately following hypoxia (Figure 1). This may be due to the delayed development in response to low oxygen availability. For example, hypoxia causes cell cycle arrest between the S and G2 phases by entering a suspended animation state (Foe and Alberts, 1985; Padilla and Roth, 2001). A recent study by Westphal et al. (2022) demonstrated that the neurogenesis of DA neurons from proliferating neural stem cells mainly occurs from 24 to 48 hpf, which overlaps with our hypoxic time window. The arrest of proliferation or TH^+^ differentiation by hypoxia may account for the delayed development of TH^+^ DA neurons shown as the reduced number of TH^+^ DA neurons (Figure 1). However, direct evidence is required to demonstrate the animation state of DA neurons by hypoxia.

Hypoxia is also known to induce apoptosis in various cell types (Tanaka et al., 2003; Honma et al., 2004; Sun et al., 2004). Interestingly, the catch-up response by HR seems to be more detrimental than the direct effects of hypoxia. The increased oxygen availability during HR accelerates overall growth to compensate for the hypoxia-restricted development; however, the concomitant ROS accumulation may trigger the P53-dependent apoptosis with HR (Johnson et al., 1996; Zhao et al., 2013; Felix et al., 2018; Shi and Dansen, 2020). Emerging evidence demonstrates that hyperoxia (i.e., a state of excess supply of O_2_ in cells and tissues) leads to an increased level of ROS and subsequent cytotoxic response in cells (Kwak et al., 2006; Auten and Davis, 2009). Therefore, the changes in oxygen availability from hypoxia to reoxygenation by HR may elevate the risk of cell damage via ROS-mediated apoptotic pathway during development. To our knowledge, the present study is the first to report not a direct effect of hypoxia but the HR-induced apoptosis of TH^+^ DA neurons in a developing vertebrate system (Figure 3).

The current work also demonstrates the differential sensitivity to HR in subpopulations of DA neurons (Figure 3). The timing or duration of hypoxia or reoxygenation by HR may be the contributing factors to differentially impact the development of DC4/5 and DC6 groups as they have different developmental stages in a specific manner, for example, the early expansion of proliferating in posterior tubercular DC4/5 and the later developing hypothalamic DC6 DA neuron groups (Mahler et al., 2010; Westphal et al., 2022); however, we cannot rule out different molecular or cellular compositions of TH^+^ DA neurons between DC4/5 and DC6 groups, leading to different responses to hypoxia or HR.

We have mainly focused on the DC4/5 DA neurons, the homologous to the A11 DAergic cell clusters of the mammalian forebrain. Several studies have shown that the DC4/5 neurons regulate spinal motor network excitability as part of developmental swimming switch to a mature adult-like swimming pattern (Ryu et al., 2007; Tay et al., 2011; Jay et al., 2015). Our data suggest that the HR-induced hypoplasia of DC4/5 TH^+^ neurons is associated with the disruption of swimming maturation in hypoxic zebrafish. It will be interesting to examine the effects of HR-induced hypoplasia of DC4/5 TH^+^ neurons on the diencephalospinal connectivity to spinal cord motor neurons and such neurons’ activity related to the swimming behavior. In addition, the present study indicates that developmental hypoxia has a long-term effect on zebrafish swimming behavior later in young adulthood. Thus, it will also be interesting to examine the effects of hypoxia or HR on the number of DC4/5 TH^+^ DA neurons in adulthood.

Another interesting aspect of our study was the effects of NAC treatment (Figures 4–6). Several studies have already shown that NAC has a protective impact on the TH^+^ DA system against 6-hydroxydopamine (6-OHDA) or ketamine-induced toxicity during development, suggesting antioxidant and neurotrophic properties of NAC protecting the DA system (Nouraei et al., 2016; Benvenutti et al., 2018; Robinson et al., 2018). We used NAC to determine potential recovery against direct hypoxic injury or indirect HR. Interestingly, the NAC with indirect HR but not with direct effects of hypoxia inhibited the expression of proapoptotic genes, including *p53* and *caspase 3*, and subsequently restored the number of DC4/5 TH^+^ DA neurons. Although the development of swimming behavior was not fully restored at 6 dpf, we observed the potential recovery in swimming development by NAC with HR. NAC has a multifaceted mechanism of action besides antioxidant effect, presenting indirect antioxidant effects by acting as a glutathione (GSH) precursor regulating proliferation and cell apoptosis, anti-inflammatory and neurotrophic effects (Aruoma et al., 1989; Circu and Aw, 2012; Samuni et al., 2013; Raghu et al., 2021). The precise mechanism of their protective effects by NAC with HR would require future study. Additionally, the impact of NAC on the ROS levels was quite interesting since it seemed to reduce the ROS in normoxic zebrafish but increase the ROS in hypoxic zebrafish. However, a thiol-containing compound of NAC can undergo autoxidation in solution generating oxygen radicals, such as superoxide free radical (Misra, 1974), which may account for the further increased ROS levels observed in HR NAC zebrafish. Therefore, we believe the neuroprotective effect of NAC found with HR may result from indirect antioxidant effects, such as GSH mediating neurotrophic effects.

## Conclusion

In summary, the current data demonstrate that developmental hypoxia significantly affects the development of DA neurons and zebrafish swimming. Indeed, HR may be more detrimental, as evidenced by the upregulation of proapoptotic genes, apoptosis, and reduced number of DC4/5 TH^+^ DA associated with impaired swimming development. Further, our data demonstrate that NAC treatment with HR restores the number of DC4/5 TH^+^ DA neurons and partially restores swimming development. Our findings may provide new insights into the abnormal motor outcomes experienced by preterm infants exposed to developmental hypoxic injury and subsequent catch-up growth by HR, as well as the potential neuroprotective effects of NAC (Ehrenkranz et al., 2006; Guellec et al., 2016; Hsu et al., 2018).

## Data availability statement

The original contributions presented in this study are included in the article/Supplementary material, further inquiries can be directed to the corresponding author.

## Ethics statement

This animal study was reviewed and approved by the Institutional Animal Care and Use Committee of the University of Scranton.

## Author contributions

J-HS and JB conceived and designed the experiments and contributed to reagents, materials, and analysis tools. J-HS, AG, and GB performed the experiments and analyzed the data. J-HS wrote the manuscript. All authors contributed to the article and approved the submitted version.

## Footnotes

1
https://imagej.net/software/fiji/
