# Supplementary material for: Hypoplasia of dopaminergic neurons by hypoxia-induced neurotoxicity is associated with disrupted swimming development of larval zebrafish
Source: Front Cell Neurosci. 2022 Sep 23;16:963037. doi: 10.3389/fncel.2022.963037 (PMC9540391; doi:10.3389/fncel.2022.963037)
Supplement: Supplementary file 1 [file Data_Sheet_1.docx]

**Supplementary Tables**

Table 1. Primer sequence for quantitative real-time PCR

| Gene Name | Gene Bank Accession No. | Primer sequence (5’-3’) |
| --- | --- | --- |
| *P53* | AF365873 | Fwd-GGGCAATCAGCGAGCAAA |
|  |  | Rev-ACTGACCTTCCTGAGTCTCCA |
| *Caspase-9* | NM152884 | Fwd-AAATACATAGCAAGGCAACC |
|  |  | Rev-CACAGGGAATCAAGAAAGG |
| *Caspase-3* | NM001048066 | Fwd-ATGAGTAGGCTTTCGGTATTGG |
|  |  | Rev- CTGTGACACTCGAACTCTCTTT |
| *Bax* | AF231015 | Fwd-GGCTATTTCAACCAGGGTTCC |
|  |  | Rev-TGCGAATCACCAATGCTGT |
| *Hatn10* | Expressed repetitive element (ERE) | Fwd- TGAAGACAGCAGAAGTCAATG |
|  |  | Rev- CAGTAAACATGTCAGGCTAAATAA |
| *Th* | BC163630.1 | Fwd-CGGCCATGTACCGATATTGT-3’ |
|  |  | Rev-GTCTCTCTGGCTCATCTGAAAG |

Table 2. Number of TH+ DA neurons 24, 48, and 72 h recovery under normoxic condition after hypoxia

|  | Normoxia | Hypoxia (24-48hpf) |
| --- | --- | --- |
| 24h recovery at 72hpf | 54.8 ± 3.3 | 10.9 ± 1.4 |
| 48h recovery at 96hpf | 66.8 ± 2.6 | 54.3 ± 3.3 |
| 72h recovery at 5dpf | 65.1 ± 1.5 | 47.9 ± 1.6 |

Table 3. Detailed swimming behavior was measured at 6dpf after the hypoxia.

|  | Normoxia | | Hypoxia (24-48hpf) | |
| --- | --- | --- | --- | --- |
|  | Dark | Light | Dark | Light |
| Total Distance (mm/10min) | 606.1 ± 72.2 | 230.4 ± 32.5 | 255.0 ± 36.5 | 141.4 ± 13.2 |
| Velocity (mm/sec) | 1.07 ± 0.12 | 0.42 ± 0.05 | 0.45 ± 0.06 | 0.27 ± 0.02 |
| Frequency of swimming (No./10 min) | 37.1 ± 5.1 | 9.2 ± 2.5 | 17.8 ± 3.9 | 6.9 ± 1.7 |

Table 4. Detailed swimming behavior was measured at 6dpf after the hypoxia with NAC exposure.

|  | Normoxia | | Hypoxia (24-48hpf) | |
| --- | --- | --- | --- | --- |
|  | Cont | NAC | Cont | NAC |
| Dark: Total Distance (mm/10min) | 660.34 ± 38.5 | 614.94 ± 37.5 | 391.6 ± 33.4 | 465.6 ± 39.8 |
| Dark: Velocity (mm/sec) | 1.68 ± 0.14 | 1.68 ± 0.17 | 1.05 ± 0.08 | 1.14 ± 0.09 |
| Dark: Frequency of swimming (No./10 min) | 42.4 ± 2.7 | 34.5 ± 3.5 | 17.8 ± 3.9 | 33.29 ± 3.3 |
| Light: Total Distance (mm/10min) | 309.5 ± 28.5 | 284.4 ± 28.6 | 258.3 ± 38.6 | 376.3 ± 65.4 |
| Light: Velocity (mm/sec) | 0.88 ± 0.10 | 0.81 ± 0.09 | 0.79 ± 0.16 | 1.02 ± 0.15 |
| Light: Frequency of swimming (No./10 min) | 20.7 ± 2.41 | 17.3 ± 2.21 | 6.8 ± 0.97 | 7.3 ± 1.38 |
