# Supplementary figures and images for: Hypoplasia of dopaminergic neurons by hypoxia-induced neurotoxicity is associated with disrupted swimming development of larval zebrafish
Source: Front Cell Neurosci. 2022 Sep 23;16:963037. doi: 10.3389/fncel.2022.963037 (PMC9540391; doi:10.3389/fncel.2022.963037)

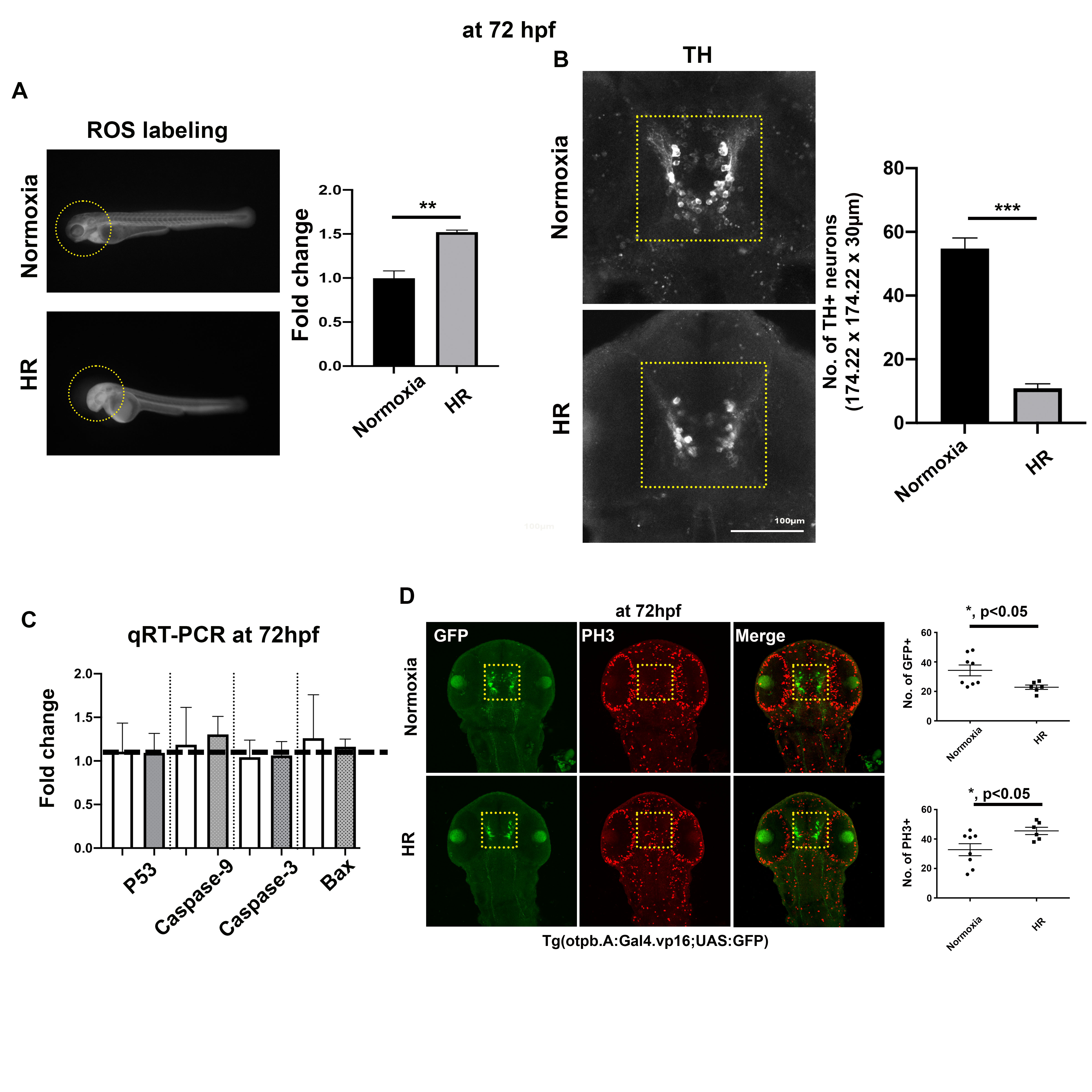

Supplement: Supplementary file 2 [file Image_1.JPEG]
